# PAIP1 regulates expression of immune and inflammatory response associated genes at transcript level in liver cancer cell

**DOI:** 10.7717/peerj.15070

**Published:** 2023-04-21

**Authors:** Jianfeng Zheng, Weiwei Fan, Xiaoyu Zhang, Weili Quan, Yunfei Wu, Mengni Shu, Moyang Chen, Ming Liang

**Affiliations:** 1 Department of Laboratory Medicine, Baoan Central Hospital of Shenzhen, The Fifth Affiliated Hospital of Shenzhen University, Shenzhen, Guangdong, China; 2 Department of Infectious Medicine, Heilongjiang Provincial Hospital, Harbin, Heilongjiang, China; 3 First Department of Infection, Second Affiliated Hospital of Harbin Medical University, Harbin, Heilongjiang, China; 4 Center for Genome Analysis, ABLife Inc., Wuhan, Hubei, China; 5 ABLife BioBigData Institute, Wuhan, Hubei, China

**Keywords:** PAIP1, Liver cancer, Gene expression, Immune response, Inflammatory response

## Abstract

Poly(A) binding protein interacting protein 1 (PAIP1) is a translation regulator and also regulate the decay of mRNA. PAIP1 has also been reported to be a marker of increased invasive potential of liver cancer. However, the roles and underlying molecular mechanism of PAIP1 in liver cancer is still unclear. Here, cell viability and the gene expression profile of liver cancer line HepG2 transfected with PAIP1 siRNA was compared with cells transfected with non-targeting control siRNA. The results showed that PAIP1 knockdown inhibited cell viability, and extensively affects expression of 893 genes at transcriptional level in HepG2 cells. Gene function analysis showed that a large number of PAIP1 up-regulated genes were enriched in term of DNA-dependent transcription and the down-regulated genes were enriched in some pathways including immune response and inflammatory response. qPCR confirmed that PAIP1 knockdown positively regulated the expression of selected immune and inflammatory factor genes in HepG2 cells. Expression analysis of TCGA revealed that PAIP1 had positive correlations with two immune associated genes IL1R2 and PTAFR in liver tumor tissue. Taken together, our results demonstrated that PAIP1 was not only a translation regulator, but also a transcription regulator in liver cancer. Moreover, PAIP1 could function as a regulatory factor of immune and inflammatory genes in liver cancer. Thus, our study provides important cues for further study on the regulatory mechanism of PAIP1 in liver cancer.

## Introduction

Liver cancer is one of the most common cancers, 75%–85% of which is hepatocellular carcinoma (HCC) (Bray et al., 2018). Hepatitis virus infection is the main risk factor for HCC (Llovet et al., 2021b). According to the latest global cancer statistics report, incidence of liver cancer ranks sixth among all cancers, and mortality rate of liver cancer has risen to third among all cancers, with about 900,000 new cases and 830,000 deaths in 2020 (Sung et al., 2021). Although the incidence and mortality of liver cancer have decreased with the promotion of hepatitis virus vaccination and improved treatment (Valery et al., 2018; Yang & Heimbach, 2020), there are controversies in the treatment of patients with intermediate and advanced liver cancer, and the prognosis of patients is still very poor (Yang & Heimbach, 2020). Therefore, it is necessary to continue to study the pathogenesis of HCC and find new targets for early diagnosis and treatment.

RNA binding proteins (RBPs) are proteins that function by binding to RNA, which could regulate transcription and post-transcriptional alternative splicing, modification, transport, translation and degradation metabolism of RNA (Hentze et al., 2018; Xiao et al., 2019). Abnormal expression or dysfunction of RBPs leads to the development of cancer and other diseases, and RBPs are potential therapeutic targets (Chatterji & Rustgi, 2018; Gebauer et al., 2021; Qin et al., 2020). It is notable that some studies have reported the roles of some RBPs in liver cancer, which are potential therapeutic targets for liver cancer (Feng et al., 2020; Silva et al., 2020; Wang et al., 2020; Yang et al., 2021). Therefore, it is necessary to continue to study the function of abnormal expression of RBPs in liver cancer and explore their mechanism of function, so as to develop personalized treatment.

Poly(A) binding protein interacting protein 1 (PAIP1) is a translation regulator that interacts with poly(A) tail binding protein (PABP) and the translation initiation factor eIF4G, which facilitate translation initiation process (Craig et al., 1998; Martineau et al., 2008; Martineau et al., 2014) and can influence the translation termination process (Ivanov et al., 2019). In addition, PAIP1 inhibits degradation of mRNA by participating in inhibiting deadenylation of mRNA (Grosset et al., 2000). Moreover, PAIP1 protein interacts with ubiquitin ligases UBR5 and WWP2 to achieve ubiquitin degradation (Lv, Zhang & Gao, 2014; Muñoz Escobar et al., 2015). As for the biology function, PAIP1 are highly expressed in many types of cancer, inducing breast cancer (Piao et al., 2018), lung adenocarcinoma (Wang et al., 2019c), pancreatic cancer (Guan et al., 2019), cervical cancer (Li et al., 2019), and gastric cancer (Wang et al., 2019b), human tongue squamous cell carcinoma (Xie et al., 2020) and gallbladder cancer (Bi et al., 2021). Knocking down PAIP1 in a variety of cancer cell lines can inhibit the proliferation and metastasis of cancer cells, and/or induce apoptosis and cell cycle arrest (Bi et al., 2021; Guan et al., 2019; Li et al., 2019; Piao et al., 2018; Wang et al., 2019b; Wang et al., 2019c; Xie et al., 2020). Mechanistically, PAIP1 inhibits lung cancer cell proliferation and epithelial-mesenchymal transition related metastasis by regulating AKT/GSK-3 *β* signaling pathway (Wang et al., 2019c). PAIP1 knockdown could decrease the expression of Ki67 and Pcna, and increased Bax/Bcl2 index and caspase-3 expression in human tongue squamous cell carcinoma (Xie et al., 2020). PAIP1 promotes gallbladder cancer through regulating expression of PLK1 (Bi et al., 2021). Results of these studies indicated that PAIP1 is a potential prognostic biomarker for cancer. It is notable that PAIP1 is highly expressed in HCC tissues compared with the normal liver tissue, and the expression of PAIP1 in HCC tissues is significantly correlated with the prognosis of patients (Kim et al., 2020). However, the roles and underlying molecular mechanism of PAIP1 in liver cancer are still unclear.

Here, to further explore the roles and regulated targets of PAIP1, we knocked down PAIP1 in HepG2 cells that was isolated from liver cancer tissue. Then, comprehensive gene expression profiles of PAIP1-knockdown cells and controls were detected by high-throughput RNA sequencing (RNA-Seq) to identify genome-wide targets regulated by PAIP1. The results showed that PAIP1 knockdown could extensively affect the expression of a large number of genes. In particular, PAIP1 knockdown inhibited expression of immune genes at mRNA level in HepG2 cells. Moreover, there were significant correlation between expression of PAIP1 and PAIP1-regulated immune genes in liver cancer tissues. These results indicated that PAIP1 cloud function as a regulatory factor of transcription, which affect expression of immune and inflammatory genes in liver cancer. Our study provides an important basis and data platform to further clarify the role of PAIP1 in mediating progress of liver cancer.

## Materials and Methods

### Cell culture and siRNA transfections

The human liver cancer cell line HepG2 and Huh7 was purchased from Procell Life Science & Technology Co., Ltd. (Wuhan, China). HepG2 cells line were cultured at 37 °C with 5% CO2 in MEM with 10% fetal bovine serum (FBS), 100 µg/mL streptomycin, 100 U/mL penicillin.

To knockdown PAIP1, three different siRNA duplexes and non-targeting control siRNA were purchased from Gemma (Suzhou, China). The sequence of these siRNA were present in Table S1. siRNA transfection of PAIP1 cells was performed using Lipofectamine 2000 (Invitrogen, Carlsbad, CA, USA) according to the manufacturer’s protocol. Transfected cells were harvested after 48 h for qPCR detection of expression of PAIP1.

### Assessment of knockdown efficiency

The total RNA of HepG2 cells transfected with different vectors was isolated using TRIzol reagent (Ambion, Austin, TX, USA). Then, cDNA was synthesized. qPCR was performed using Bestar SYBR Green RT-PCR Master Mix (DBI Bioscience, Shanghai, China) on the Bio-Rad S1000. GAPDH (glyceraldehyde-3-phosphate dehydrogenase) was used as a reference gene for assessing the efficiency of PAIP1 knockdown. The primers used for qPCR are listed in Table S1. The expression of PAIP1 was then normalized to GAPDH using 2^−ΔΔCT^.

### Western blotting

HepG2 cells were lysed in ice-cold wash buffer (1 × PBS, 0.1% SDS, 0.5% NP-40 and 0.5% sodium deoxycholate) supplemented with a protease inhibitor cocktail (Roche, Basel, Switzerland) and incubate on ice for 30 min. Samples were boiled for 10 min in boiling water with 1X SDS sample buffer and separated on 10% SDS-PAGE. With TBST buffer (20 mM Tris-buffered saline and 0.1% Tween-20) containing 5% non-fat milk power for 1 h at room temperature, membranes were incubated with primary antibody: PAIP1 antibody (1:1,000, ABclonal, Wuhan, China), GAPDH (1:2,000, ABclonal, Wuhan, China) and then with HRP-conjugated secondary antibody. Bound secondary antibody was detected using the enhanced chemiluminescence (ECL) reagent (170506, BioRad, Hercules, CA, USA).

### Cell viability analysis

A MTT assay was used to evaluate cell viability according to a previous study (Wang et al., 2019a). Cultured cells were pretreated with 20 µl MTT solution. The treated cells were cultured under the same conditions for 30 min. Then, the medium was removed. Next, 0.15 ml dimethylsulfoxide (DMSO) was used to solubilize the resulting formazan crystal. An ELISA reader was used to detect the optical density at 490 nm.

### Library preparation and sequencing

The total RNA of HepG2 cells was extracted with TRIZOL (Ambion, Austin, TX, USA) and purified with two phenol-chloroform. Then, the purified total RNA was treated with RQ1 DNase (Promega, Madison, WI, USA) to remove DNA. Further purified RNA was detected for the absorbance at 260 nm/280 nm (A260/A280) using Smartspec Plus (BioRad, Hercules, CA, USA) to assess the quality and quantity. Agarose gel electrophoresis (1.5%) was performed to assess the integrity of the purified RNA.

For RNA-seq library preparation, 1 µg RNA was used for each sample. Oligo(dT)-conjugated magnetic beads (Invitrogen, Carlsbad, CA, USA) were used to purify and concentrate polyA mRNAs from the total RNA sample. The purified and concentrated RNA sample was fragmented. End-repair and 5′  adaptor ligation of fragmented RNA were performed. Then, the RNA were reversely transcribed using primers harboring 3′  adaptor sequence and randomized hexamer for cDNAs. The cDNAs were further amplified and purified. The purified cDNAs were stored at −80 °C until sequencing.

According to the manufacturer’s instructions, high-throughput sequencing libraries were prepared. Pair-end Sequencing (151-bp) were conducted by Illumina HiSeq4000 system in a commercial company (ABLife Inc., Wuhan, China).

### Clean and alignment of raw sequencing data

First, raw sequencing reads with more than 2-N bases were discarded. Then, FASTX-Toolkit (Version 0.0.13) was used to trim the adaptors and low-quality bases from raw reads. The resulting reads less than 16nt were further discarded. Tophat2 were used to align the clean reads to the GRch38 genome allowing 4 mismatches (Li et al., 2013). Then, uniquely mapped reads of each gene were used to calculate the FPKM (paired-end fragments per kilobase of exon per million fragments mapped) (Trapnell et al., 2010).

### Differentially Expressed Genes (DEG) analysis

FPKM was used to evaluate the expression level of genes. edgeR software was used to identify the differentially expressed genes (DEGs) (Robinson, McCarthy & Smyth, 2010) with a threshold for the fold change (fold change ≥2 or ≤0.5) and false discovery rate (FDR < 0.05).

### Functional enrichment analysis of DEGs

Overrepresentation analysis (ORA) were performed for the upregulated or downregulated DEGs to detect gene set enrichment by Gene Ontology (GO) and reactome pathway analysis was used to predict the gene function and calculate the functional category distribution frequency (Xie et al., 2011). Reactome functions as an archive of biological processes and as a tool for discovering unexpected functional relationships (Fabregat et al., 2018). The hypergeometric test and Benjamini–Hochberg FDR controlling procedure were used to define the enrichment of each pathway (corrected *p*-value < 0.05 or FDR < 0.05).

### Expression validation of DEGs by qPCR

Quantitative real-time PCR (qPCR) of selected DEGs was conducted to elucidate the validity of the RNA-seq data. GAPDH was used as a reference gene. Primers for qPCR was presented in Table S2. qPCR was conducted on the same RNA samples which was used for RNA-seq. PCR reaction conditions were as follows: denaturing for 10 min at 95 °C, then 40 cycles including denaturation for 15 s at 95 °C, with annealing and extension for 1 min at 60 °C. Three technique replicates were performed for PCR amplification of each sample.

### Analysis of TCGA (The Cancer Genome Atlas) data

The RNA-seq data of liver cancer were downloaded from TCGA database (https://www.cancer.gov/). Expression of PAIP1 and PAIP1-reguated DEGs in liver cancer sample were analyzed.

### Analysis of the Human Protein Atlas (HPA)

The Human Protein Atlas stores plenty of information about thousands of proteins regarding their expression and distribution in a variety of cancer tissues. Using this database, we analyzed the differential expression of PAIP1 protein level between normal liver tissues and liver cancer tissues.

### Statistical analysis

Statistical analysis was performed using R (v3.1.3). qPCR data of the two groups were compared by an unpaired two-tailed *t*-test at *P* values <0.05. The qPCR data were presented as the mean ± standard deviation (SD). Pearson’s correlation test was used to assess the relationship between expression of PAIP1 and PAIP1-regulated DEGs in liver cancer tissues from TCGA at *P* values <0.05.

## Results

### PAIP1 was overexpressed in liver cancer tissues and associated with poor prognosis

**Figure 1:**
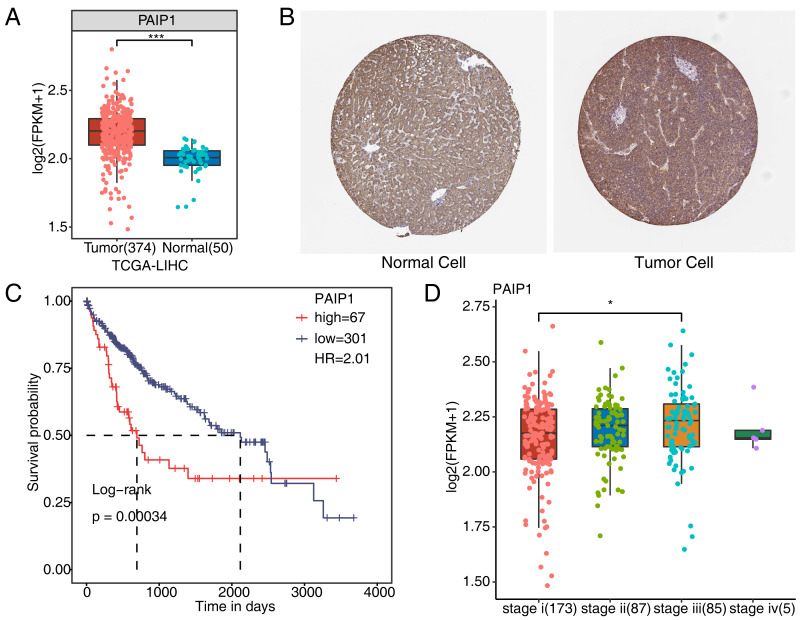
Expression of PAIP1 in clinical samples of liver cancer of TCGA and the Human Protein Atlas database. (A) PAIP1 has a high expression at mRNA level in tumor tissue based on the TCGA samples. (B) PAIP1 has a high expression at protein level in tumor tissue based on the Human Protein Atlas database samples. (C) Patients with high expression of PAIP1 had a lower survival rate compared to patient with low PAIP1 expression. (D) PAIP1 has a relative high expression in tissue of advanced liver cancer.

To verify whether PAIP1 was overexpressed in liver cancer tissues, we detected the expression of PAIP1 in liver cancer. We analyzed the expression of PAIP1 in liver cancer sample of the TCGA and the Human Protein Atlas database, the results showed that the expression of PAIP1 was obviously up-regulated in liver cancer tissues compared to normal tissues at both transcript and protein level (Figs. 1A and 1B). Moreover, the expression of PAIP1 was correlated with the prognosis of patients with liver cancer. Compared to patients with low expression of PAIP1, patients with high expression had shorter overall survival (Fig. 1C). Additionally, compared to early-stage (stage i) liver cancer, PAIP1 showed a relative higher expression in advanced liver cancer (stage iii) (Fig. 1D). Thus, PAIP1 might be a potential prognostic factor in liver cancer.

### PAIP1 knockdown inhibited cell viability and extensively regulated gene expression at transcriptional level in HepG2 cells

**Figure 2:**
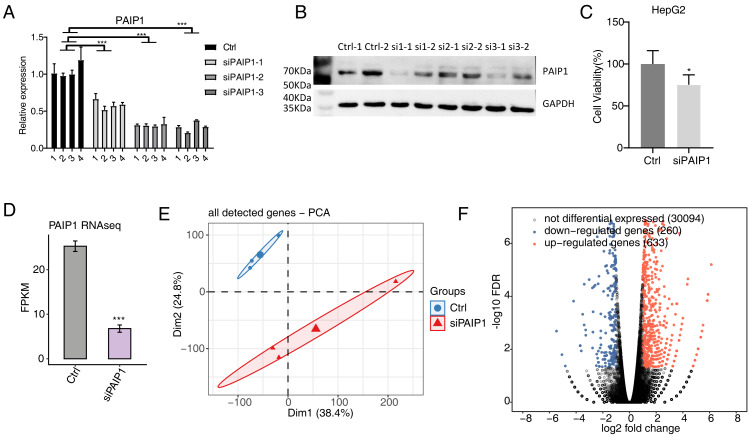
Effects of PAIP1 knockdown on cell viability and the gene expression profile of HepG2 cells. (A) PAIP1 knockdown were validated by qPCR. siPAIP1-1, siPAIP1-2, and siPAIP1-3 were three different PAIP1 siRNAs. The number 1, 2, 3, and 4 were biological replicates of each siRNA. (B) PAIP1 knockdown were validated by WB. si1, si2, and si3 were three different PAIP1 siRNAs. The number si1-1 (si2-1 or si3-1) and si1-2 (si2-2 or si3-2) were biological replicates of each siRNA. (C) PAIP1 knockdown was validated by RNA-seq. (D) PAIP1 knockdown inhibit the cell proliferation. (E) PCA analysis showed differences in gene expression profile between control and PAIP1 knockdown samples. (F) Differential expressed genes (DEGs) were identified after PAIP1 knockdown. Red and blue dots represent upregulated and downregulated genes in the volcano plot, respectively. For qPCR, GAPDH was used as the reference gene. Student’s *t* test was performed to compare PAIP1-knockdown and control cells with significance set at a *P* value of less than 0.05. ^∗^ *P* < 0.005, ^∗∗∗^ *P* < 0.001.

To explore the regulated targets of PAIP1 in liver cancer, the expression of PAIP1 was knocked-down in HepG2 cells by transfecting vector with three different PAIP1 siRNAs (siPAIP1-1, siPAIP1-2, and siPAIP1-3) (Table S1). Compared with cells being transfected with non-targeting control siRNA (NC), all three PAIP1 siRNAs effectively reduced the mRNA and protein expression of PAIP1 (Figs. 2A and 2B). Notably, siRNA3 had the best knockdown efficiency (Figs. 2A and 2B), which was used to knock-down PAIP1 in HepG2 cells for cell viability and RNA-seq. As is shown, the cell viability of HepG2 cells were inhibited by PAIP1 knockdown (Fig. 2C).

Then, RNA-seq was used to detect the gene expression profiles of PAIP1 knockdown with siRNA3 and control HepG2 cells. Both siPAIP1 and control HepG2 cells had three biological replicates. There were six RNA-seq samples (siPAIP1_1st, siPAIP1_2nd, siPAIP1_3rd, Ctrl_1st, Ctrl_2nd, Ctrl_3rd). After cleaning raw sequence data, at least 38.8 million pair-end reads were obtained for each sample. After mapping those clean reads to the human genome, more than 66.8 million uniquely mapped reads of each sample were used for gene expression further gene expression analysis (Table S3).

FPKM were calculated to represent the expression level of each gene. The results showed that there were unique 30,705 genes with FPKM >0 and unique 12,862 genes with FPKM >1 in at least one sample (Tables S4 and S5). PAIP1 knockdown were further verified by FPKM values of this gene in HepG2 cells (Fig. 2D). According to FPKM values of each expressed genes, the six samples that were used for Principal Component Analysis (PCA). As shown, there was a clear separation of siPAIP1 and control samples with three biological replicates in a group (Fig. 2E). This result demonstrated that PAIP1 knockdown obviously changed the gene expression profile of HepG2 cells.

**Figure 3:**
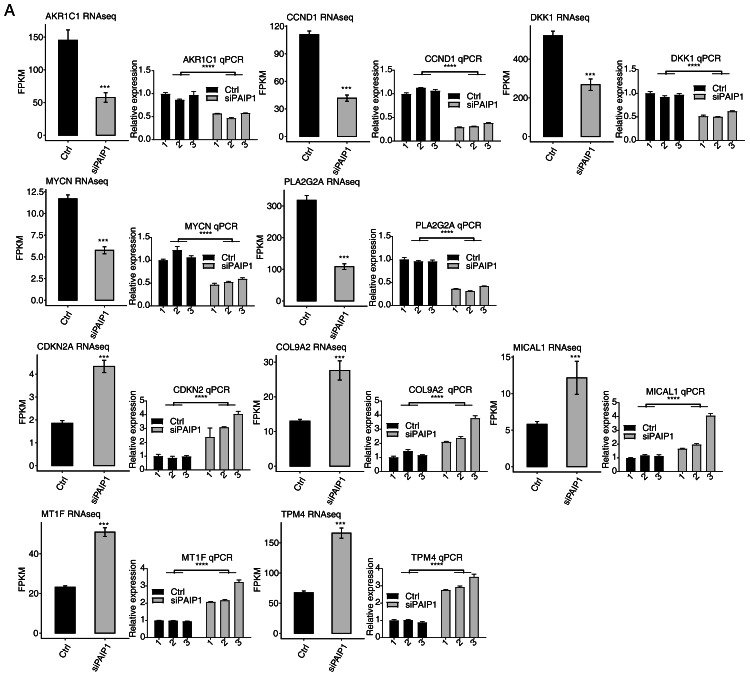
Validation of PAIP1-knockdown regulated genes in HepG2 cells. Relative expression levels of ten PAIP1-knockdown regulated DEGs by RNA-seq (FPKM) (left) and qPCR (right). For qPCR, GAPDH was used as the reference gene. Student’s *t* test was performed to compare PAIP1-knockdown and control cells with significance set at a *P* value of less than 0.05. ^∗∗∗^ *P* < 0.001, ^∗∗∗∗^ *P* < 0.0001.

Then, DEGs between the siPAIP1 and control cells were identified by the edgeR to further compare the gene expression profile. A total of 633 upregulated and 260 downregulated DEGs were identified between siPAIP1 and control cells (Fig. 2F). All the DEGs were listed with detailed information including FPKMs and fold changes (Table S6). To verify the effect of PAIP1-knockdown on the expression of these DEGs, qPCR was conducted to quantify the changes in mRNA levels of these genes after PAIP1 knockdown in HepG2 cells. Ten DEGs were selected for qPCR analysis, including five upregulated genes and five downregulated genes. All selected DEGs with FPKM >1 in at least one sample. The results showed that all selected DEGs showed a significant increase or decrease after PAIP1- knockdown in HepG2 cells, which was in agreement with the RNA-seq analysis (Fig. 3). These results showed that PAIP1 extensively regulates gene expression in HepG2 cells.

**Figure 4:**
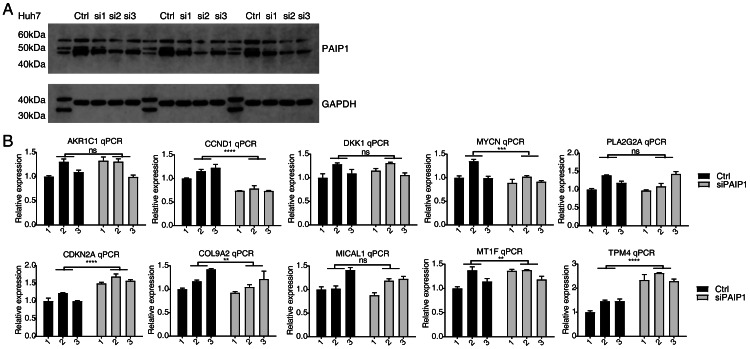
Validation of PAIP1-knockdown regulated genes in Huh7 cells. (A) PAIP1 knockdown were validated by WB. si1, si2, and si3 were three different PAIP1 siRNAs. Three different ctrl, si1, si2, and si3 were biological replicates of each siRNA. (B) Relative expression levels of ten PAIP1-knockdown regulated DEGs by qPCR. For qPCR, GAPDH was used as the reference gene. Student’s *t* test was performed to compare PAIP1-knockdown and control cells with significance set at a *P* value of less than 0.05. ^∗∗^ *P* < 0.01, ^∗∗∗^ *P* < 0.001, ^∗∗∗∗^ *P* < 0.0001.

In addition, the expression of PAIP1 was knocked-down in another liver cancer cell Huh7 by transfecting vector with same three different PAIP1 siRNAs (siPAIP1-1, siPAIP1-2, and siPAIP1-3) (Table S1). Compared with cells being transfected with non-targeting control siRNA (NC), all three PAIP1 siRNAs effectively reduced protein expression of PAIP1 (Fig. 4A). qPCR was conducted to quantify the changes in mRNA levels of the same ten identified DEGs in HepG2 cells. The results showed that five genes showed a significant increase or decrease after PAIP1-knockdown in Huh7 cells (Fig. 4B), which was in agreement with that of HepG2 cells. These results showed that PAIP1 could regulate expression of some genes in both HepG2 and Huh7 cells.

### PAIP1 knockdown inhibited immune and inflammatory genes in HepG2 cells

**Figure 5:**
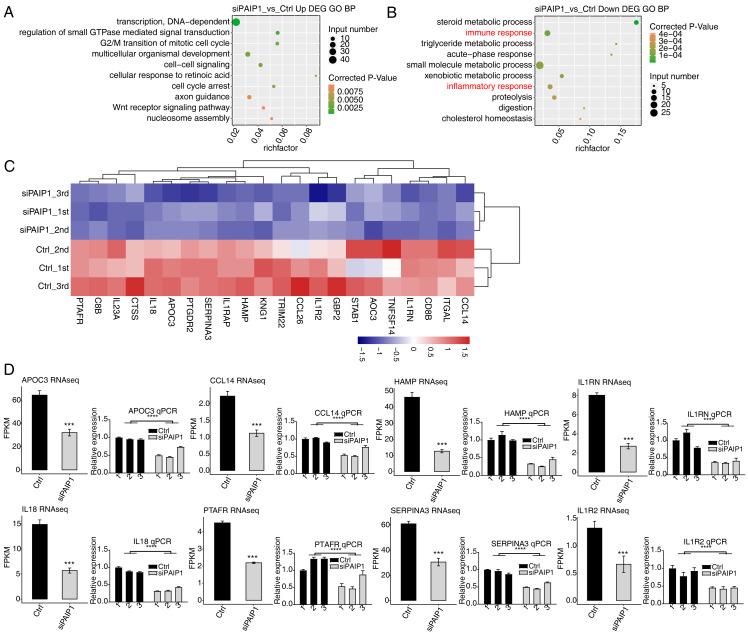
Functional analysis of DEGs after PAIP1 knockdown in HepG2 cells. (A) The top 10 GO biological processes terms of PAIP1-knockdown upregulated genes. (B) The top 10 GO biological processes terms of PAIP1-knockdown downregulated genes. (C) Heatmap and hierarchical cluster of DEGs between control and PAIP1 knockdown samples. FPKM values are log2-transformed and then median-centred by each gene. (D) Relative expression levels of ten PAIP1 negatively regulated DEGs by RNA-seq (FPKM) (left) and qPCR (right). For qPCR, GAPDH was used as the reference gene. Student’s *t* test was performed to compare PAIP1-knockdown and control cells with significance set at a *P* value of less than 0.05. ^∗∗∗^ *P* < 0.001.

To reveal the potential roles of DEGs, GO analysis was performed to annotate 633 upregulated DEGs. GO analysis was divided into three categories: biological process (GO-P), molecular function (GO-F) and cellular component (GO-C). The results revealed 170 upregulated genes annotated with 45 GO-P, terms (Table S7). The top 10 GO-P terms include DNA-dependent transcription, regulation of small GTPase mediated signal transduction, G2/M transition of mitotic cell cycle, multicellular organismal development, cell–cell signaling, cellular response to retinoic acid, cell cycle arrest, axon guidance, Wnt receptor signaling pathway, and nucleosome assembly (Fig. 5A). These results indicated that PAIP1 knockdown increased the expression of many transcription factors and cell cycle associated genes.

To further reveal the roles of downregulated DEGs, GO analysis was performed to annotate those DEGs. The results revealed 124 downregulated genes annotated with 33 GO-P terms (Table S8). The top 10 GO-P terms include steroid metabolic process, immune response, triglyceride metabolic process, acute-phase response, small molecule metabolic process, xenobiotic metabolic process, inflammatory response, proteolysis, digestion, and cholesterol homeostasis (Fig. 5B). These results indicated that PAIP1 positively regulates the expression of many genes associated with immune response and inflammatory response.

To further explore the potential function of PAIP1-regulated genes, reactome pathway analysis was performed. The results showed that the upregulated DEGs were enriched in terms such as HDACs deacetylate histones, DNA methylation, and PRC2 methylates histones and DNA (Table S9). The downregulated DEGs were enriched in many immune terms such as interleukin-10 signaling, signaling by interleukins, interleukin-18 signaling Table S10). These results also indicated that PAIP1 potentially regulated the expression of immune response and inflammatory response genes.

Therefore, we selected some genes related to immune response and inflammatory response in DEGs and did hierarchical clustering of standardized FPKM values in HepG2 cells. The results indicated that there was a significant separation between the PAIP1-knockdown sample and the control sample, and the three replicate data sets were highly consistent (Fig. 5C). To verify the effect of PAIP1-knockdown on the expression of these DEGs, qPCR was conducted to quantify the changes in mRNA levels of these genes after PAIP1 knockdown in HepG2 cells. Eight DEGs were selected for qPCR analysis, including APOC3, SERPINA3, HAMP, IL18, IL1RN, PTAFR, CCL14 and IL1R2. All selected DEGs with >1 in at least one sample. The results showed that all selected DEGs showed a significant decrease after PAIP1- knockdown in HepG2 cells, which was in agreement with the RNA-seq analysis (Fig. 5E). These results indicated that PAIP1 positively regulates the expression of immune and inflammatory genes in HepG2 cells.

### PAIP1 showed a positive or negative correlation with immune and inflammatory genes in liver cancer tissues

**Figure 6:**
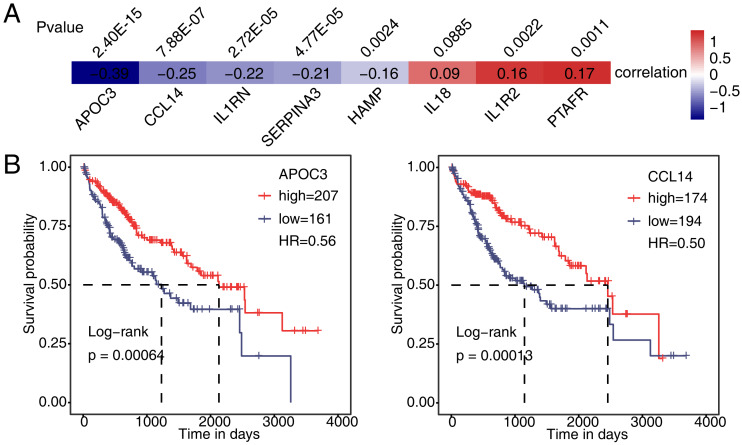
Validation of PAIP1-regulated genes in liver cancer sample of TCGA. (A) Seven DEGs showed a positive or negative correlation with PAIP1 in clinical samples of liver cancer. (B) Patients with high expression of APOC3 and CCL14 had a higher survival rate compared to patient with low expression.

Then, we analyzed the correlation between expression of PAIP1 and eight immune and inflammatory response genes in liver cancer tissues from the TCGA. The results showed that the expression of PAIP1 had a significant negative correlation (*P* <  0.05) with that of APOC3, CCL14, IL1RN, SERPINA3, and HAMP in liver cancer tissues (Fig. 6A). There were significantly positive correlation (*P* < 0.05) between PAIP1 expression and PTAFR and IL1R2 (Fig. 6A). Thus, PAIP1 would positively regulated PTAFR and IL1R2 in both HepG2 cells and liver cancer tissues (Figs. 5E and 6A). In addition, we analyzed the relationship between the expression of these PAIP1 regulated genes with the prognosis of patients with liver cancer. Compared to patients with low expression of APOC3 and CCL14, patients with high expression had better overall survival (Fig. 6B). Therefore, these results indicated that PAIP1 potentially regulated immune and inflammatory genes in liver cancer tissue.

## Discussion

PAIP1 is a RNA binding protein, by which PAIP1 participates in regulation of translation (Craig et al., 1998; Ivanov et al., 2019; Martineau et al., 2008; Martineau et al., 2014) and mRNA decay (Grosset et al., 2000). PAIP1 are highly expressed in many types of cancer (Bi et al., 2021; Guan et al., 2019; Li et al., 2019; Piao et al., 2018; Wang et al., 2019b; Wang et al., 2019c; Xie et al., 2020), inducing liver cancer (Kim et al., 2020). However, the genome-widely regulated targets and mechanism of PAIP1 in liver cancer is still unclear. Here, our study showed that PAIP1-knockdown inhibited cell viability, and broadly regulated expression of many transcription factors and immune and inflammatory genes at the transcript level in HepG2 cells. The results indicated that PAIP1 would be a regulator factor of transcription, which affect the expression of immune and inflammatory genes in liver cancer. Therefore, this study provides important cues for further study on the regulatory mechanism of PAIP1 in liver cancer.

In recent years several studies show that PAIP1 are highly expressed in different type of cancer and are potential prognostic biomarker (Li et al., 2019; Piao et al., 2018; Wang et al., 2019b; Wang et al., 2019c). In fact, a previous study reports that PAIP1 is also highly expressed in cancer issues of hepatocellular carcinoma, and is a potential prognostic marker of patients (Kim et al., 2020). In this study, the gene expression profiles and clinical information of liver hepatocellular carcinoma samples from TCGA were downloaded to analyze the expression of PAIP1 in all the tumor and normal liver tissues. The results demonstrated that PAIP1 had a higher expression in tumor than normal liver tissue, and patients with high PAIP1 expression in tumor had a poor overall survival rate. Therefore, two independent set of data suggest that PAIP1 is a prognostic marker of hepatocellular carcinoma. However, previous published study does not reveal how PAIP1 functions in hepatocellular carcinoma. Thus, it will be desirable to explore how PAIP1 functions in liver cancer.

Previous studies reported that PAIP1 knockdown inhibit the cell viability in a variety of cancer cell lines (Bi et al., 2021; Guan et al., 2019; Li et al., 2019; Piao et al., 2018; Wang et al., 2019b; Wang et al., 2019c; Xie et al., 2020). Our study showed that PAIP1 knockdown can inhibit cell viability of liver cancer, which is consistent with previous studies. Recently, several studies report that PAIP1 could regulated expression of AKT/GSK-3 *β* signaling pathway genes in lung cancer, Ki67, Pcna, Bax/Bcl2 and caspase-3 in tongue squamous cell carcinoma, and PLK1 in gallbladder cancer, respectively (Bi et al., 2021; Wang et al., 2019c; Xie et al., 2020). In this study, PAIP1 was knocked down in HepG2 cells and genome-wide regulated targets of PAIP1 were identified by RNA-seq. Differential expressed genes showed that PAIP1 knockdown promoted expression of 633 genes and inhibited expression of 260 genes in HepG2 cells. This result indicated that PAIP1 broadly regulated the gene expression at transcript level in HepG2 cells. As we known, our study firstly identified the genome-widely regulated targets of PAIP1 in liver cancer, which is different from previous study of PAIP1 in hepatocellular carcinoma (Kim et al., 2020). It is notable that PAIP1 knockdown decreased the expression of many immune and inflammatory factors in HepG2 cells. In fact, inflammation and immune response play important roles in development and progression of hepatocellular carcinoma, and revealing the molecular mechanism of immune and inflammation is of great significance in the immunotherapy of liver cancer (Keenan, Fong & Kelley, 2019; Llovet et al., 2021a; Yu, Ling & Wang, 2018). This results indicated that that PAIP1 may play a role as immune regulator in liver cancer. In addition, a recently study reported that aberrant hyperexpression of the RNA binding protein FMRP mediates immune evasion of tumor cell (Zeng et al., 2022). We speculated that PAIP1 may play a role as immune regulator and promote the immune evasion of liver cancer cell, which partially interpreted that high PAIP1expression had shorter overall survival. Thus, exploring the molecular mechanisms that PAIP1 regulate the immune and inflammatory factors will be meaningful to immunotherapy of liver cancer in future work.

Based on the results of RNA-seq and qPCR of our study HepG2 cells, PAIP1 positively regulates the expression of all eight selected immune and inflammatory factors. However, only two genes (PTAFR and IL1R2) were significantly positively correlated with PAIP1 expression in liver tumor samples from TCGA, which is same to regulation direction of PAIP1 on these genes in HepG2 cells. We speculated that tumor tissues were highly cellular heterogeneous, and a gene may had different expression level in tumor cell from tumor microenvironment (TME) cells. In fact, single-cell RNA sequencing of liver tumor tissue has reveal the heterogeneity of malignant cells and immune cells in hepatocellular carcinoma (Ma et al., 2019; Yang et al., 2020; Yang & Heimbach, 2020; Zhang et al., 2019). At least, our study showed that PAIP1 could potential positively regulate the expression of PTAFR and IL1R2 both in HepG2 cells and liver tumor tissues. PTAFR is high expressed in cervical cancer samples when compared with normal cervical tissue, which protect tumor cells from radiation-induced cell death (da Silva-Junior et al., 2018). Increased levels of IL1R2 are involved in the initiation and progression of human gastric cancer, and IL1R2 might be an immunotherapeutic target (Yuan et al., 2021). It could be speculated that both PTAFR and IL1R2 would play important roles in liver cancer. Thus, more work could be conducted to explore the roles of PTAFR and IL1R2 in liver cancer.

## Conclusions

In conclusion, this study demonstrated that PAIP1 was high expressed in liver cancer tissue, and PAIP1 knockdown inhibit cell viability and extensively regulate the expression of a number of genes in HepG2 cells. This result indicated that PAIP1 was not only a translation regulator, but also a transcription regulator in liver cancer. In particular, we first revealed that PAIP1 promote expression of many genes associated with immune and inflammatory response in HepG2 cells. Thus, PAIP1 may play a role as immune regulator in liver cancer. Our study provides important cues for further study on the regulatory mechanism of PAIP1 in liver cancer.

##  Supplemental Information

10.7717/peerj.15070/supp-1 Supplemental Information 1 Information of siRNA used in this study

10.7717/peerj.15070/supp-2 Supplemental Information 2 Primers that were used for qPCR

10.7717/peerj.15070/supp-3 Supplemental Information 3 Summary of sample names, description, the RNA-seq sequencing information and mapping results in each sample

10.7717/peerj.15070/supp-4 Supplemental Information 4 Gene expression level (FPKM)

10.7717/peerj.15070/supp-5 Supplemental Information 5 Human genes detected by different FPKM cut off

10.7717/peerj.15070/supp-6 Supplemental Information 6 Differential expression of genes between siPAIP1 and control

10.7717/peerj.15070/supp-7 Supplemental Information 7 GO biology process terms of PAIP1-knockdown upregulated genes

10.7717/peerj.15070/supp-8 Supplemental Information 8 GO biology process terms of PAIP1-knockdown downregulated genes

10.7717/peerj.15070/supp-9 Supplemental Information 9 Reactome pathways of PAIP1-knockdown upregulated genes

10.7717/peerj.15070/supp-10 Supplemental Information 10 Reactome pathways of PAIP1-knockdown downregulated genes

10.7717/peerj.15070/supp-11 Supplemental Information 11 Raw data for qPCR validation

10.7717/peerj.15070/supp-12 Supplemental Information 12 Raw data for Western Blotting

## Additional Information and Declarations

### DNA Deposition

The following information was supplied regarding the deposition of DNA sequences: The raw data of RNA-seq are available in the NCBI Gene Expression Omnibus: GSE185866.

### Data Availability

The following information was supplied regarding data availability: The raw measurements are available in the Supplementary File.
