# Supplementary material for: PAIP1 regulates expression of immune and inflammatory response associated genes at transcript level in liver cancer cell
Source: PeerJ. 2023 Apr 21;11:e15070. doi: 10.7717/peerj.15070 (PMC10124545; doi:10.7717/peerj.15070)
Supplement: Supplemental Information 12 [file peerj-11-15070-s012.docx]

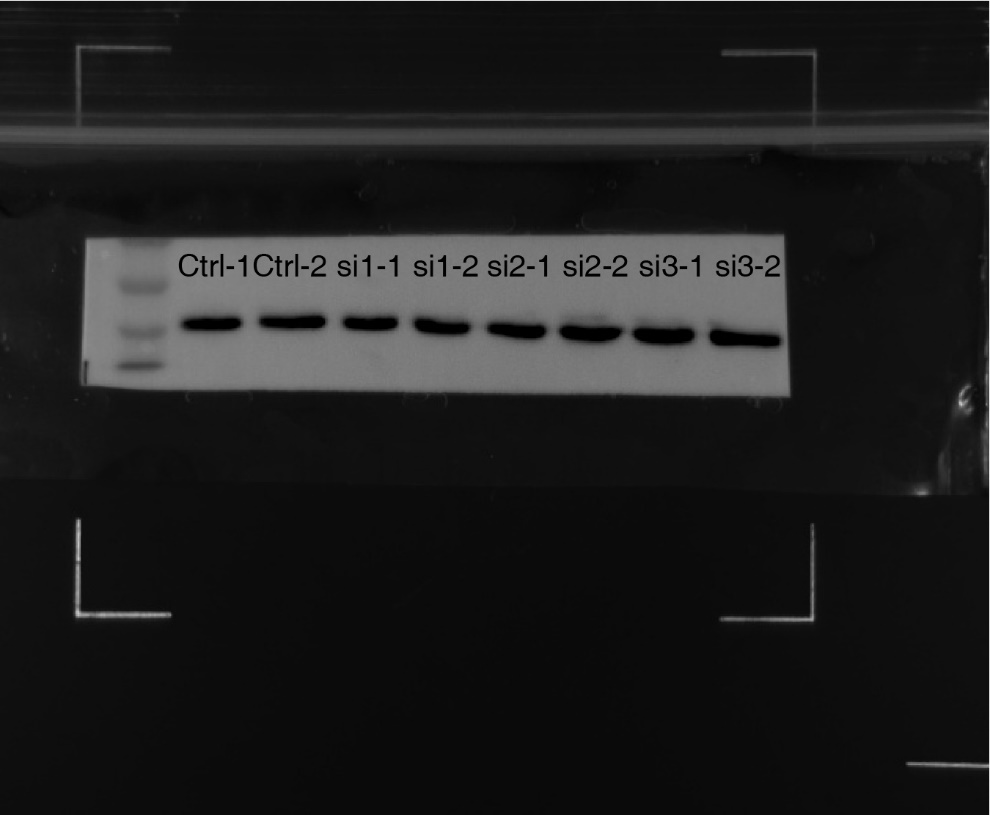


Photograph of GAPDH band in HepG2.


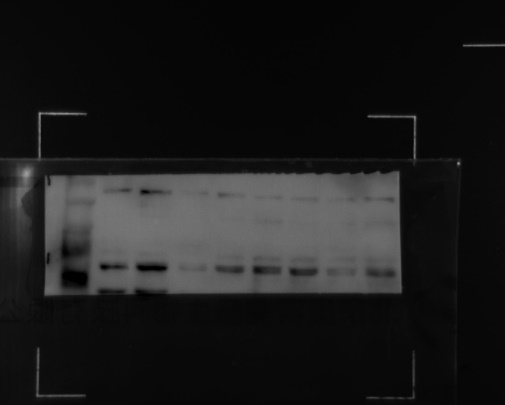


Photograph of PAIP1 band in HepG2.


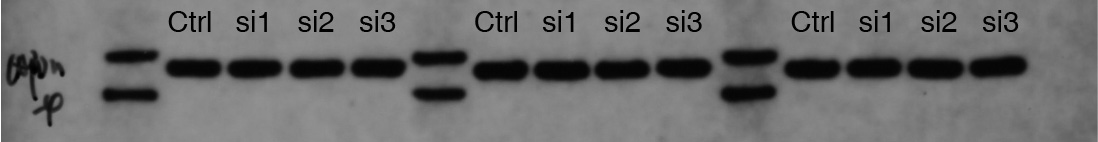


Photograph of GAPDH band in Huh7.


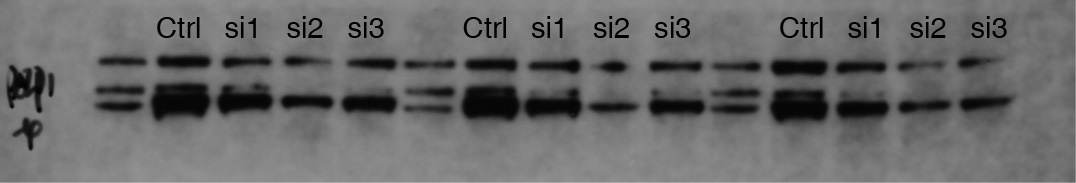


Photograph of PAIP1 band in Huh7.
